# Small GTPase Ran: Depicting the nucleotide-specific conformational landscape of the functionally important C-terminus

**DOI:** 10.3389/fmolb.2023.1111574

**Published:** 2023-01-16

**Authors:** Janka Czigleczki, Pedro Tulio de Resende Lara, Balint Dudas, Hyunbum Jang, David Perahia, Ruth Nussinov, Erika Balog

**Affiliations:** ^1^ Department of Biophysics and Radiation Biology, Semmelweis University, Budapest, Hungary; ^2^ Department of Medical Genetics and Genomic Medicine, School of Medical Sciences, University of Campinas—UNICAMP, Campinas, Brazil; ^3^ Inserm U1268 MCTR, CiTCoM UMR 8038 CNRS—Université Paris Cité, Paris, France; ^4^ Laboratoire et Biologie et Pharmacologie Appliquée, Ecole Normale Supérieure Paris-Saclay, Gif-sur-Yvette, France; ^5^ Computational Structural Biology Section, Frederick National Laboratory for Cancer Research in the Cancer Innovation Laboratory, National Cancer Institute, Frederick, MD, United States; ^6^ Department of Human Molecular Genetics and Biochemistry, Sackler School of Medicine, Tel Aviv University, Tel Aviv, Israel

**Keywords:** Ran, small GTPase, conformational switch, C-terminus, conformational search, molecular dynamics, normal modes, aMDeNM

## Abstract

The small GTPase Ran is the main regulator of the nucleo-cytoplasmic import and export through the nuclear pore complex. It functions as a molecular switch cycling between the GDP-bound inactive and GTP-bound active state. It consists of a globular (G) domain and a C-terminal region, which is bound to the G-domain in the inactive, GDP-bound states. Crystal structures of the GTP-bound active form complexed with Ran binding proteins (RanBP) show that the C-terminus undergoes a large conformational change, embracing Ran binding domains (RanBD). Whereas in the crystal structures of macromolecular complexes not containing RanBDs the structure of the C-terminal segment remains unresolved, indicating its large conformational flexibility. This movement could not have been followed either by experimental or simulation methods. Here, starting from the crystal structure of Ran in both GDP- and GTP-bound forms we show how rigid the C-terminal region in the inactive structure is during molecular dynamics (MD) simulations. Furthermore, we show how MD simulations of the active form are incapable of mapping the open conformations of the C-terminus. By using the MDeNM (Molecular Dynamics with excited Normal Modes) method, we were able to widely map the conformational surface of the C-terminus of Ran in the active GTP-bound form, which allows us to envisage how it can embrace RanBDs.

## 1 Introduction

Ran (Ras-related nuclear), which belongs to the Ras superfamily of small GTPases, is the main regulator of nucleo-cytoplasmic import and export through the nuclear pore complex (NPC) (Gorlich, 1998; Joseph, 2006). It controls cell cycle progression by regulating the of microtubule polymerization and mitotic spindle formation (Paci et al., 2021) playing an essential role in tumor progression and metastasis (Boudhraa et al., 2020).

As other small GTPases, Ran is a molecular switch cycling between GDP-bound cytosolic inactive- and GTP-bound nucleus-located active states. The GDP/GTP alternation is controlled by guanine nucleotide exchange factors (GEFs), which stimulate the intrinsically slow GDP/GTP exchange, and the GTPase activating proteins (GAPs), which stimulate the GTP hydrolysis.

The function of Ran is based on the localization of the RanGEF regulator of chromosome condensation1 protein (RCC1) only in the nucleus (Ohtsubo et al., 1989; Seki et al., 1996) and of RanGAP only in the cytoplasm, which create a Ran-GTP gradient across NPC (Gorlich and Kutay, 1999). The import of Ran-GDP into the nucleus is carried out by nuclear transport factor 2 (NTF2), where the complex dissociates (Gorlich et al., 1996). Binding of RCC1 to Ran-GDP in the nucleus stimulates the GDP/GTP exchange. Ran-GTP fuels the export of exportin and the cargo molecule, as well as the export of the nuclear transport receptor (NTR) back to the cytosol. In the cytosol Ran-GTP is hydrolyzed by RanGAP in the presence of Ran Binding Protein (RanBP1/RanBP2) (Bischoff and Gorlich, 1997).

**FIGURE 1:**
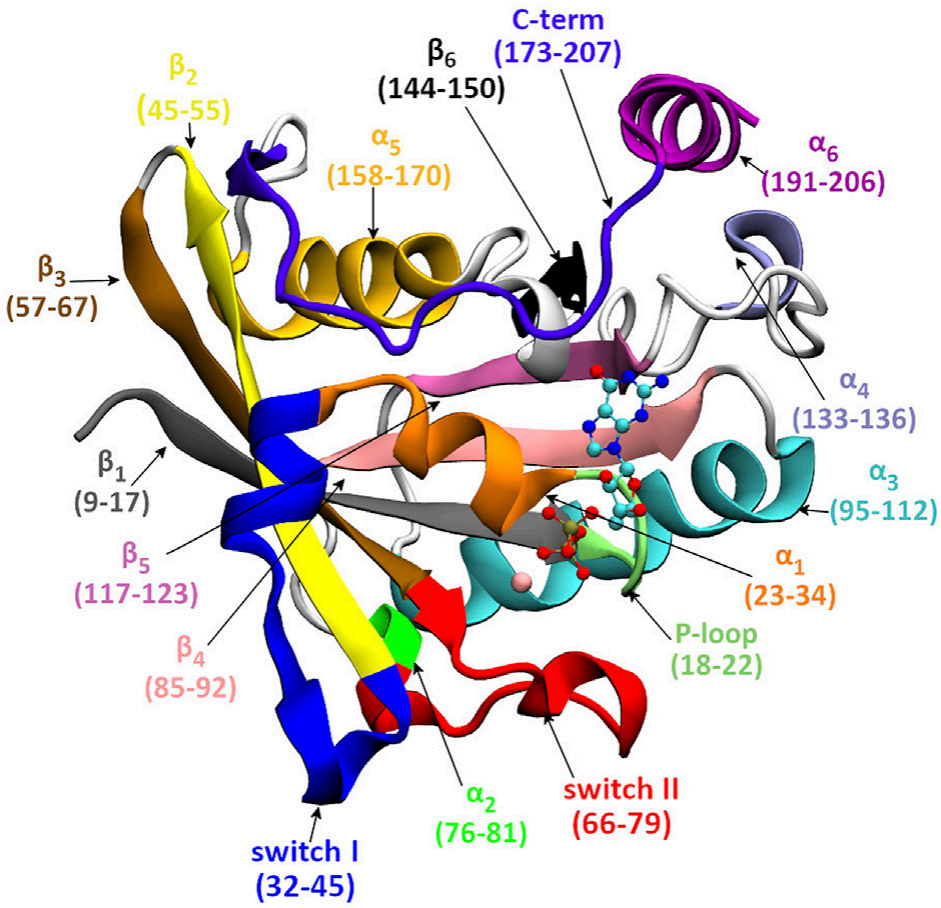
The secondary structure elements of ran. The GDP is shown as CPK, Mg^2+^ as vdW.

**FIGURE 2:**
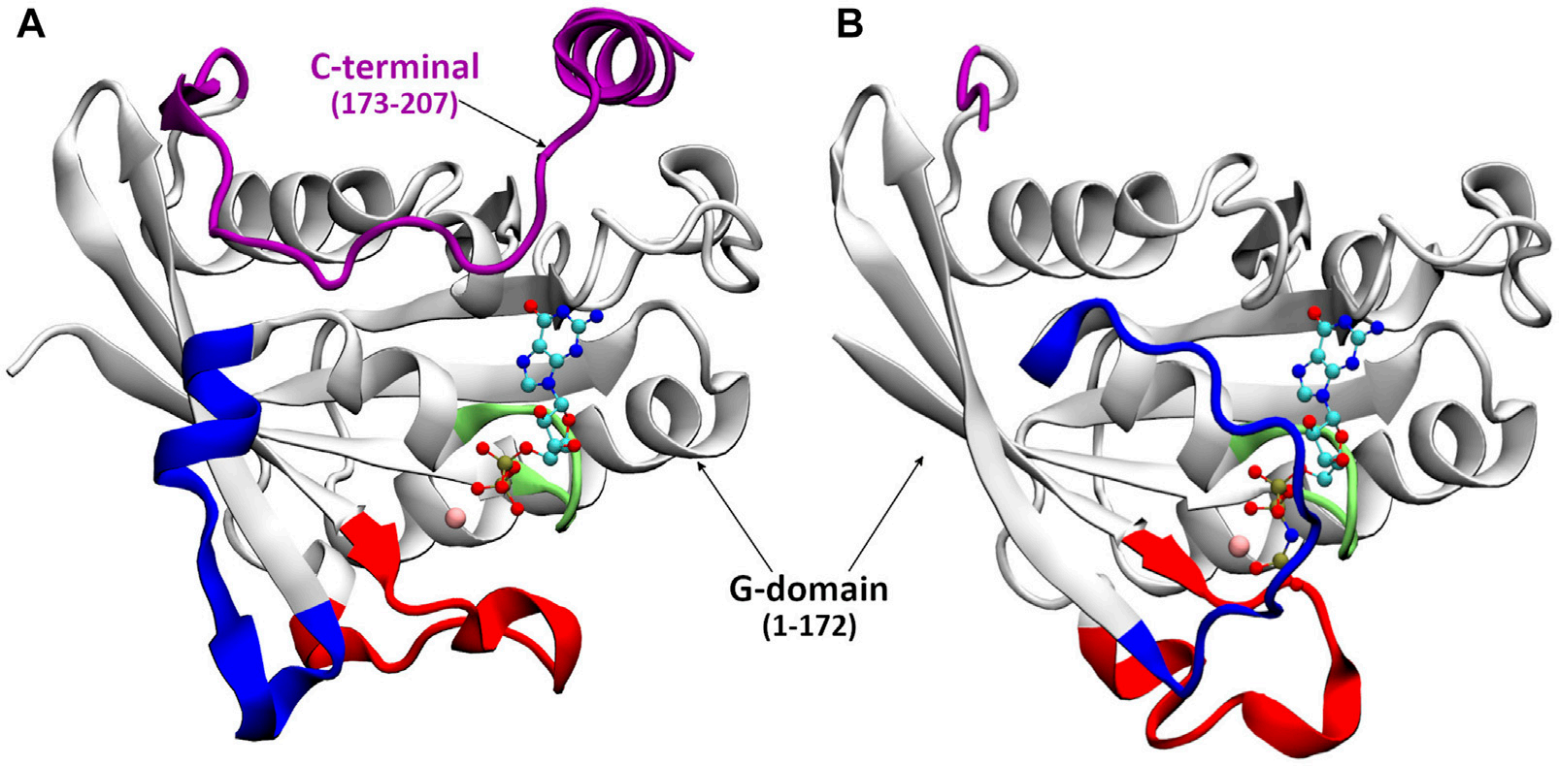
The crystal structure of Ran-GDP **(A)** and Ran-GTP **(B)**. Different structural elements are color coded: P-loop (lime), switch I (blue), switch II (red), C-terminal (purple), the GDP/GTP is denoted by CPK, Mg^2+^ by pink vdW.

The full-length structure of Ran-GDP (see Figure 1), is composed of a G-domain (GTP binding domain, residue 1–172) and a C-terminus (residue 173–216) which terminates in a unique acidic tail (DEDDDL) (Scheffzek et al., 1995). The G-domain—as in other GTPases—contains the phosphate-binding loop (P-loop) that, together with the Mg^2+^ ion, stabilizes the nucleotide binding; and two critical motifs, Switch I and Switch II, which upon nucleotide exchange undergo a major conformational change allowing the interaction with downstream partners (Figure 2).

As shown in Figures 1, 2, in the inactive GDP-bound form, the C-terminus is wrapped around the G-domain, known in the literature as being stabilized by the interaction of the C-terminal acidic tail with the ‘basic patch’ of the G-domain (Vetter et al., 1999b). The standalone full-length Ran-GTP structure has not been determined but it has been crystalized in complex forms. In crystal structures, when analogues of Ran-GTP form complexes with Ran Binding Proteins (RanBP) (Vetter et al., 1999b; Seewald et al., 2002; Sun et al., 2013; Rudack et al., 2015), the C-terminus is embracing RanBP. In contrast, macromolecular complexes without RanBP contain only the G-domain of Ran-GTP (Vetter et al., 1999a; Renault et al., 2001; Forwood et al., 2008; Monecke et al., 2009; Guttler et al., 2010), indicating that the C-terminus is flexible and its structure could not been solved.

In this article, we report how nucleotide-specific the flexibility of the C-terminus is. In the inactive form, besides the interactions known in literature, we identify the interactions that keep the C-terminal helix rigidly bound to the G-domain. We show that classical molecular dynamics (MD) simulations do not efficiently map the active, open conformations of the C-terminus. By using adaptive molecular dynamics with excited normal modes (aMDeNM) method, we were able to depict conformations that could not have been assessed either experimentally or with classical MD simulations.

## 2 Materials and methods

MD and further developed version of MDeNM (Costa et al., 2015), the “adaptive MDeNM” (aMDeNM) (Resende-Lara et al., 2022) simulations were carried out on GDP- and GTP-bound Ran.

The starting coordinates of the human Ran-GDP were taken from the crystal structure with PDB ID 5CIQ (Rudack et al., 2015). Although it has been suggested that the DEDDDL (residues 210–216) acidic C-terminal tail interacts with the basic patch of the G-domain (residues 139–142) (Vetter et al., 1999b), the acidic tail has not been crystalized (indicating its mobility). To avoid introducing any bias, we have not completed the crystal structure with the acidic tail.

For human Ran-GTP, the crystal structure with PDB ID 5CLL (Rudack et al., 2015) was used as a starting point. Since this structure does not contain the C-terminal helix, the missing C-terminal part (residues 185–208) was completed using 5CLQ (which is a Y39A mutant) (Rudack et al., 2015) by overlapping the C-terminal segment of residues 176–184. The GTP analogue GDP-BeF was modified to GTP.

aMDeNM simulations and analysis were performed with CHARMM (Brooks et al., 2009) using the CHARMM all-atom additive force field C36 (Best et al., 2012), while conventional MD simulations were carried out with NAMD (Phillips et al., 2020) using the same CHARMM force field above. The GDP/GTP parameters were taken from our previous studies (Dudas et al., 2021).

The structures were solvated using CHARMM-GUI(Jo et al., 2008; Jo et al., 2014). For Ran-GDP, a rectangular box containing TIP3 water molecules was built extending 14 Å in all directions from the surface of the molecule. For Ran-GTP, since a large conformational change is expected, a rectangular water box with 39 Å in all directions from the surface of the protein was created. The NaCl concentration was set to 0.15 M in both cases.

For energy calculations, the dielectric constant was set to 1. The Particle Mesh Ewald (PME) method was used to calculate the electrostatic interactions with a grid spacing of 1 Å or less having the order of 6; the real space summation was truncated at 12.0 Å, and the width of the Gaussian distribution was set to 0.34 Å^−1^. The van der Waals (vdW) interactions were reduced to zero by “switch” truncation operating between 10.0 and 12.0 Å.

Solvated systems were energy minimized with gradually decreasing harmonic restraints applied to Cartesian coordinates of the heavy atoms: first, steepest descent was used with the harmonic force constant of these restrains decreased every 500 steps having successively 10, 1, and 0.1 kcal/mol/Å^2^, followed by 200 conjugate gradient steps with a force constant of 0.1 kcal/mol/Å^2^. Unrestrained minimization was then applied for 100 steps with steepest descent, 200 steps with conjugate gradient, and 1,000 steps with the adopted basis Newton-Raphson method. The energy-minimized structures were heated and equilibrated at 300 K for 200 ps in an NVT ensemble, followed by a 5 ns NPT run at a pressure of 1 atm. Langevin dynamics was used with a damping coefficient of 1 ps^−1^, a piston oscillation period of 50 fs, and a piston oscillation decay time of 25 fs. The integration time step was set to 2 fs.

For the production run three independent 200 ns long MD simulations were performed for both systems with different initial velocity distributions, starting from the final structure of the 5 ns equilibration run. The parameters for the 200 ns run were identical to those of the 5 ns equilibration.

### 2.1 aMDeNM simulations

aMDeNM (Resende-Lara et al., 2022) simulations were carried out in order to efficiently map the conformational space of the C-terminus.

The normal modes necessary for the aMDeNM simulations were calculated in vacuum by considering the final structures resulting from the 5 ns equilibration run for both GDP- and GTP-bound structures. First, the energy of the structures was minimized using the steepest descent method, the harmonic force constant decreasing every 1,000 steps, adopting the values 10, 1, 0.1, and 0 kcal/mol/Å^2^, followed by 50,000 steps of adopted basis Newton-Raphson method. Thereafter, the normal modes were calculated using the VIBRAN module of CHARMM.

For the closed Ran-GDP structures, no normal mode that would open the C-terminus was found, such aMDeNM could not have been applied.

For Ran-GTP, based on their root-mean-square fluctuation (RMSF) contribution, 4 low frequency normal modes were taken. The final structure of the 5 ns equilibration run was considered as initial structure for aMDeNM simulations.

Randomized linear combinations of the four normal modes were generated, providing the excitation directions. In order to ensure an exhaustive search of the conformational space, the new excitation directions were compared to the previously accepted ones and were only kept if the root-mean-square deviation (RMSD) value—between the structures displaced by 1 Å along the mode combinations—was greater than 1.15 Å. A total of 183 aMDeNM replica simulations were carried out corresponding to each of the retained different normal mode combinations. These excitation directions were then used to kinetically excite the systems in successive small MD simulations of 0.2 ps with a sustained kinetic energy injection of 1.25 kcal/mol. As the trajectory evolves and the structure undergoes large conformational changes, the initial excitation directions are no longer valid since they were computed with the initial structure as reference. Therefore, the method adapts the excitation direction allowing the exploration of new regions of the conformational space that would not be accessed. The adaptation is done by taking into account two factors: a given displacement of the system along the excitation direction; and the extent that the effective trajectory has deviated from the theoretical one (a projection of the displacement of the system along the excitation vector). If the displaced distance along the excitation direction is ≥0.5 Å and the effective displacement deviated more than 60° from the theoretical displacement, the excitation direction is updated. The new directions correspond to the difference of the average position of the structures obtained in the last excitation and the starting position of this simulation. Each period of excitation-relaxation yields a given conformation; therefore, considering that 200 excitations per replica were generated, we obtained 36,600 structures.

As seen in Supplementary Figure S1, the RMSD values in all MD simulations reached their plateau indicating that the systems reached stable conformations. Even replicas of aMDeNM, despite the energy injection and short simulations time, reach relatively stable states.

An alternative to aMDeNM simulations to better grasp the conformational variability consists of methods that update the normal modes multiple times during the simulations, such as ClustENM (Kurkcuoglu et al., 2016) and CoMD (Gur et al., 2013), which use elastic network models. One major advantage of using aMDeNM is to continuously change the global motion within the complete physical force field of the molecular system during the MD simulation, not requiring further calculations of NM directions that are usually done in a vacuum. Other advantages of aMDeNM are fully described in the article of Resende-Lara et al. (Resende-Lara et al., 2022).

## 3 Results and discussion

**FIGURE 3:**
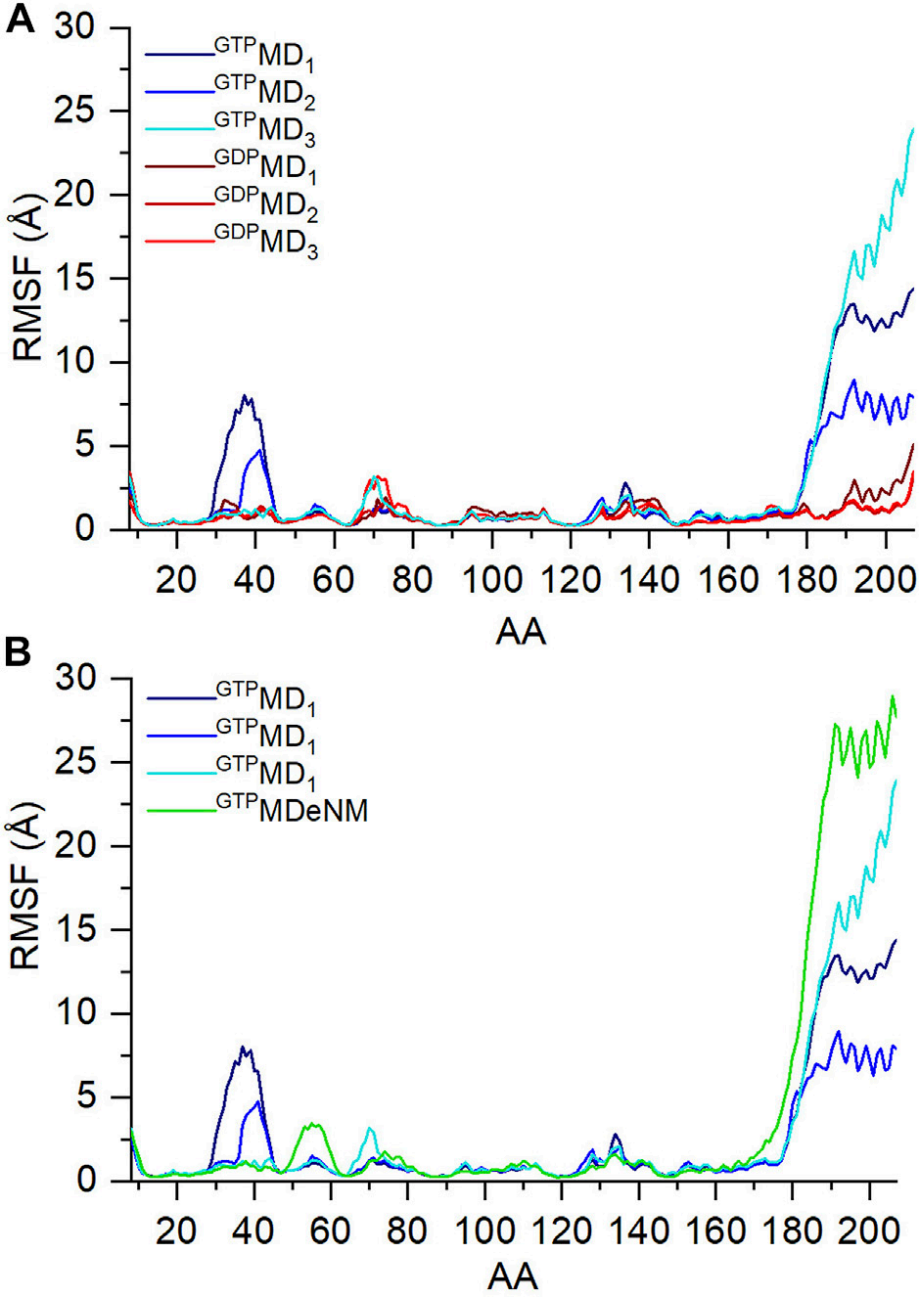
RMS fluctuation values of C_α_ atoms during the three independent MD simulations of **(A)** ran-GDP (shades of red) and ran-GTP (shades of blue); and **(B)** of ran-GTP during MD (shades of blue) and aMDeNM (green) simulation.

To have an overview of the dynamics, the RMSF of atomic displacements per residue of the GDP- and GTP-bound Ran are shown in Figure 3. As expected from experimental data, the analysis of the MD data (Figure 3A), shows that the fluctuation of the C-terminus is increases in the GTP bound form. But unlike the K-Ras behavior (Dudas et al., 2020), in the GTP bound state, Ran exhibits large fluctuation of the Switch I region compared to the GDP bound state. This could be interpreted by the conformational change undergone by Switch I: from an ordered α-helix/β-turn conformation in the GDP-bound structure (Figure 2A) and turning to a disordered loop structure in the GTP-bound state (Figure 2B). Switch II, like K-Rras, rigidifies upon GDP/GTP exchange.

In Figure 3B we compare the fluctuation of Ran-GTP obtained with the two simulation methods, MD and aMDeNM. The C-terminus indeed shows higher fluctuation when aMDeNM was applied compared to MD simulations, suggesting that the C-terminus maps a wider conformational space when using the aMDeNM.

As mentioned in *Materials and methods* part, aMDeNM could not been performed on the GDP-bound Ran, since no normal mode was found that would open the C-terminus of the closed Ran-GDP structure. This fact also indicates that the Ran-GDP is a stable, “rigid” structure.

The other difference between the MD and aMDeNM fluctuation of Ran-GTP is the missing Switch I fluctuation for the aMDeNM calculation. This could have been caused by the fact that the four normal modes that were chosen for the aMDeNM calculations focus on the movements of the C-terminus.

### 3.1 Mapping the possible conformations of the C-terminus

**FIGURE 4:**
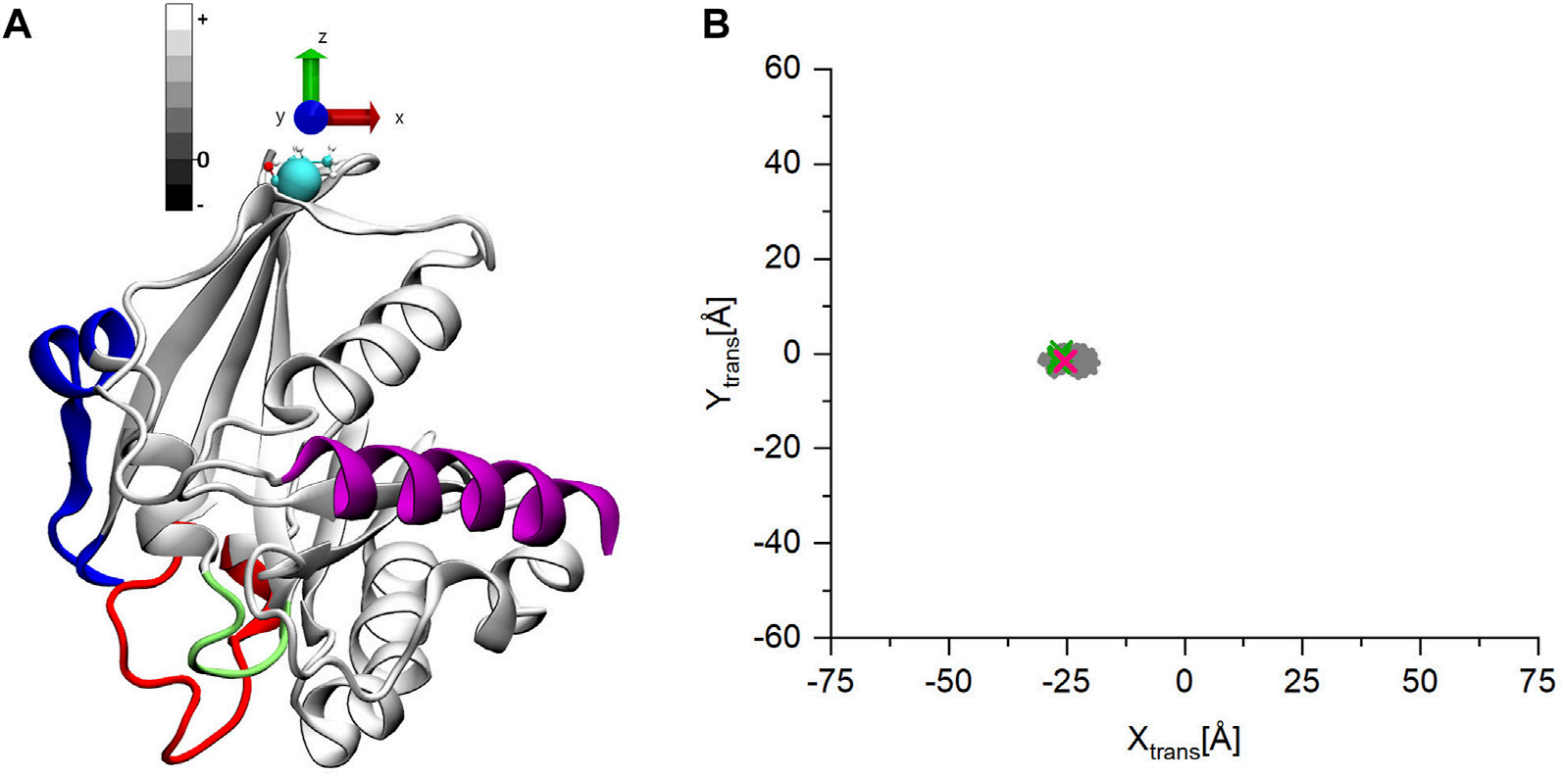
**(A)** The starting structure for the MD simulations of ran-GDP after the coordinate transformation. **(B)** The coordinates of the center of mass (COM) of the C-terminal helix on the x-y plane. The points are gray-scale-coded depending on their z coordinate values. The pink mark denotes the starting structure, the green the coordinates are of the other experimentally determined structures.

To follow in detail the conformational movements of the C-terminus with respect to the G-domain, we performed a coordinate transformation throughout the trajectories such that the origin of the coordinate system is placed at the base of the C-terminal tail (residue 177) (indicated by a cyan sphere on Figure 4A). Furthermore, the z-axis is aligned along the largest moment of inertia of the G-domain, pointing away from the G-domain with the x-y plane being perpendicular to it. Further, we calculated the center of mass (COM) of the C-terminal α -helix on this transformed coordinate system. In this way the z values of COM represent how the C-terminus moves away (+) or approaches (−) the G-domain along the z-axes, while the x-y values show on which side of the G-domain the C-terminus can be found.

Figure 4B shows how the C-terminus is trapped in a stable position for all three Ran-GDP MD simulations. The starting open structure (Figure 4A) is denoted by a pink mark in Figure 4B. The COM of the C-terminal α-helix of the known Ran-GDP experimental structures are also shown by green marks on the figure, which also indicates how well-defined the position of the C-terminus is with respect to the G-domain in these structures (Supplementary Movie S1).

**FIGURE 5:**
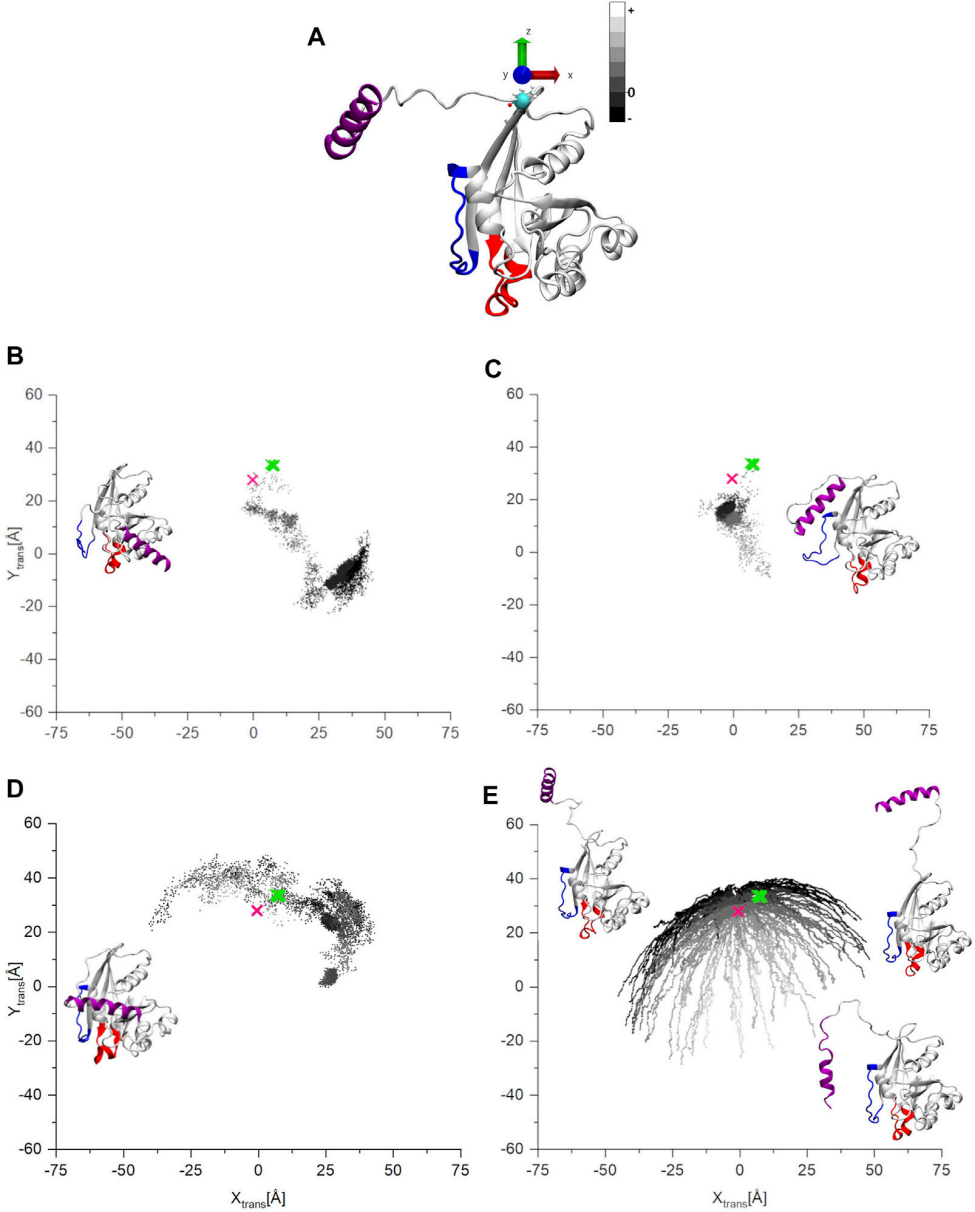
**(A)** The starting structure for the MD and aMDeNM simulations of Ran-GTP after the coordinate transformation. **(B–D)** The coordinates of the COM of C-terminal helix on the x-y plane of the three parallel MD simulations, **(E)** the result of the aMDeNM simulation. The points are gray-scale-coded depending on their z coordinate values. The pink mark denotes the starting structure, the green the coordinates are of the other experimentally determined structures. In the inset, the final structures of the three MD simulations are represented. While for the aMDeNM part **(E)** some representative structures are shown.

Figure 5 shows the positions of the C-terminus for Ran-GTP during of the three MD and for the aMDeNM trajectories. As previously, the pink mark on the graphs indicates the starting structure for the simulations (Figure 5A). As we can see, during all three MD simulations (Figures 5B–D), the open starting structure, after mapping a more confined (simulation 1, 2—Figures 5B, C) or a more extensive (simulation 3—Figure 5D) part of the conformational space, finds its way to get stabilized by closing at different locations on the surface of the G-domain (Supplementary Movie S2).

In contrast to all three MD simulations, the aMDeNM results of Ran-GTP (Figure 5E) show that the C-terminus maps a wide range of open conformations on different sides of the G-domain (Supplementary Movie S3).

As mentioned previously, the C-terminus of Ran-GTP could be crystalized only in complexes with Ran-binding proteins (Seewald et al., 2002; Sun et al., 2013), always embracing the Ran-binding domain. The green marks in the figures denote the COM coordinates of the of the C-terminal helices of these crystal structures. The concentrated position of the C-terminus COMs show that the experimentally determined structures are always constrained to a similar conformation. Complementary to this, the aMDeNM simulation results do show how the C-terminus of Ran-GTP can map a wide conformational space, being able to interact, and then having a stabilized 3D structure with the Ran-binding domains.

### 3.2 Interactions of the C-terminus with the G-domain

To have a clearer picture of the interactions that trap a given C-terminus conformation during the MD simulations, the pairwise interaction energies of the amino acids constituting the G-domain and the C-terminus were calculated. Energy values were obtained as the sum of pairwise non-bonded electrostatic and vdW energy contributions, using CHARMM.

**FIGURE 6:**
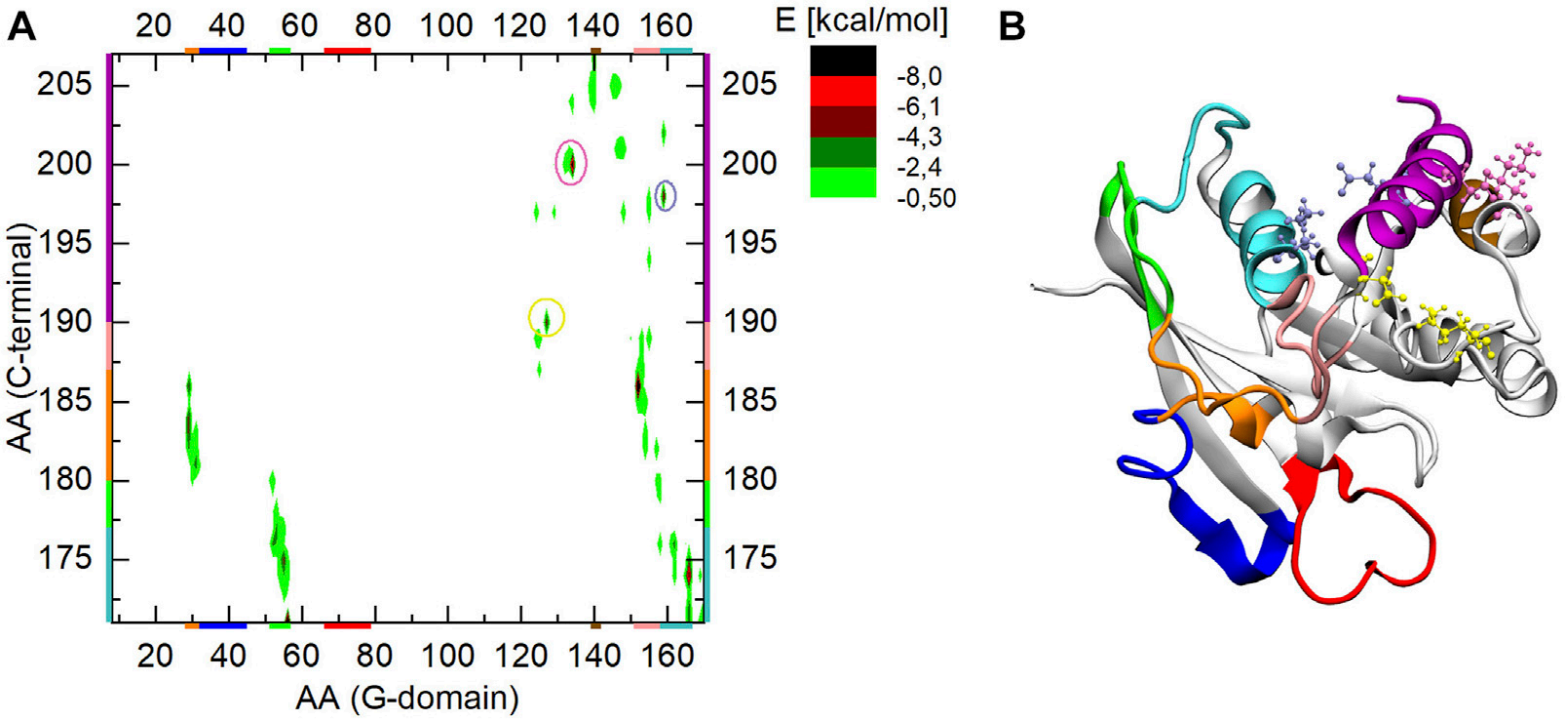
**(A)** The interaction energy map between the G-domain and the C-terminus during MD simulation of Ran-GDP, **(B)** 3D structure of Ran-GDP. The interacting regions are color-coded both along the axes or encircled on the graph and mapped with identical coloring onto the 3D structure. The encircled parts of the **(A)** part highlight the strong ionic interactions of the C-terminal helix denoted by similar color CPKs on the **(B)** part of the figure.

In order to have a reference point, Figure 6A shows the interaction energy map of the three independent Ran-GDP simulations. To more easily identify the interacting elements, the final Ran-GDP structure of the simulation is shown in Figure 6B, with a color-coding similar to that on the axes of the energy plot.

Besides the well-known C-terminal end—G-domain basic patch (residues 139–142, denoted by brown) (Vetter et al., 1999b) interaction, we found the following elements stabilizing the inactive conformation.

i) residues 171–180 of the C-terminal loop (denoted by cyan and green) are intercalated between helix α5 and the sheet β2, stabilized through residues Leu174/Arg166 forming a backbone-sidechain H-bond, and Glu175/Asn55, Phe176/His53 and Asp171/Arg56 interacting *via* electrostatic and ionic interactions respectively.
ii) the orange part of the C-term loop (residues 181–186) interacts with α1 (also denoted by orange) through Ala181/Leu31 and Ala183/Arg29 *via* two H-bond, while the pink part of the loop (residues 187–189) is in closed proximity to loop β6-α5 showing an ionic interaction between 186Glu/152Lys.
iii) the C-terminal helix is attached to the G-domain *via* three strong ionic interactions: one at its N-terminal end involving Asp190/Lys127 (encircled by yellow and denoted by yellow CPK), at 198Glu/159Lys (encircled by violet and denoted by violet CPK) and at 200Asp/134Lys (encircled by mauve and denoted by mauve CPK).

**FIGURE 7:**
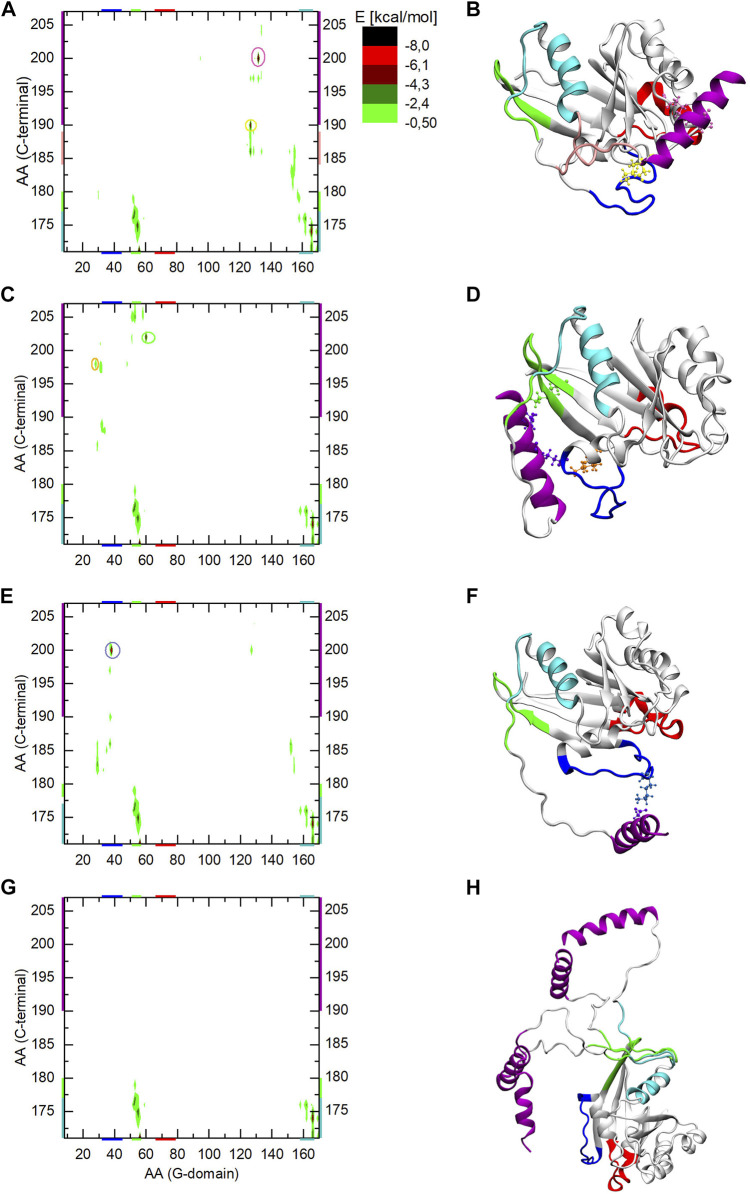
The interaction energy map between the G-domain and the C-terminal of Ran-GTP during the three parallel MD simulation (part **A,C,E** of the Figure) and during aMDeNM simulation (part **G** of the Figure). The interacting regions are color-coded both along the axes or encircled on the graph, and mapped with identical coloring onto the corresponding end structures of the given simulation (part **B,D,F, and H** of the Figure). The reported energy values are statistical averages over given MD or aMDeNM simulations.

Since in all three Ran-GTP MD simulations the C-terminus reached different sides of the G-domain, the interaction energies are also shown separately in Figures 7A, C, E corresponding to the three MD simulations. The respective final structures of the simulations are shown in Figures 7B, D, F.

In all three MD simulations, the N-terminal end of the C-terminus shows an interaction pattern similar to the Ran-GDP case, remaining intercalated between α5 and β2 of the G-domain.

In the case of the first MD simulation the C-terminal loop interacts with the β6-α5 loop of the G-domain (colored pink) and the C-term helix is stabilized by the ionic interactions between 190Asp/127Lys (encircled and denoted by yellow CPK), and 200Asp/132Lys (encircled and denoted by mauve CPK).

In the second MD simulation, the C-terminal helix gets attached to the other part of the G-domain in the proximity of β2 and β3 (denoted by green), by forming ionic interactions between 202Glu/60Lys (orange CPK) and 198Glu/28Lys (green CPK).

In the third MD simulation, the C-terminus gets attached to Switch I *via* the ionic interaction between 200Asp/38Lys.

By learning about the interactions listed above, we can also interpret the different RMSF behavior of Switch I during the three simulations (Figure 3A): we note that it diminishes in the third simulation compared to the previous two, indicating that the ionic interaction between the C-terminus and Switch I confines the switch in a given conformational state, while in the other two simulations its movement is not restricted and explores different conformations.

The interaction energy map of the aMDeNM simulation (Figure 7G) shows that, as for all previously studied structures, the N-terminal end of the C-terminal loop (denoted by cyan and green) remains intercalated between α5 and β2 of the G-domain, showing a similar interaction energy pattern. No other interactions can be seen in the map, indicating that the C-terminal helix maps different open conformations, as it is shown in Figure 7H and Figure 5E.

By comparing the C-terminus conformations reached by the different simulation methods to the X-ray structures of the known Ran complexes we can note that in two out of the three MD simulations, the final conformation of the C-terminus overlaps with the RanBP binding site to the G-domain. Knowing that the starting structure of the Ran-GTP simulations was taken from the RanBP1 complex, this clearly shows how constrained the conformational mapping of the MD simulations were. The third MD simulation does not seem to overlap with known biologically relevant surfaces of the G-domain. In contrast, during the aMDeNM simulations the C-terminus maps a wide range of open conformations that do not interact with the G-domain. These open conformations could enable the C-terminus to interact with the molecular counterparts i.e. RanBPs.

## 4 Conclusion

By using two simulation approaches (MD and aMDeNM) we were able to characterize the nucleotide-dependent dynamical behavior of the C-terminal end of Ran, the biological role of which has been reported by numerous experimental articles.

In the inactive GDP-bound form the C-terminus end stays rigidly attached to the G-domain, auto inhibiting the effector binding site. Besides the interactions known in the literature, we identified three intense ionic interactions that keep the C-terminal helix rigidly bound to the G-domain, namely, Asp190/Lys127, 198Glu/159Lys and 200Asp/134Lys.

Thus, the MD simulations are shown to be incapable of efficiently depicting the active, open conformations of C-terminus in the active GTP-bound form of Ran. By using the aMdeNM method, we were able to map conformations that could not have been assessed either experimentally or with classical MD simulations. The wide variety of the obtained C-terminus conformations allows us to envisage how Ran-GTP is capable of interacting with its macromolecular partners.

## Data Availability

The raw data supporting the conclusions of this article will be made available by the authors, without undue reservation.

## References

[B1] Best R. B. Zhu X. Shim J. Lopes P. E. Mittal J. Feig M., (2012). Optimization of the additive CHARMM all-atom protein force field targeting improved sampling of the backbone φ ψ and side-chain χ(1) and χ(2) dihedral angles. J. Chem. Theory Comput 8 (9), 3257–3273. 10.1021/ct300400x 23341755 PMC3549273

[B2] Bischoff F. R. Gorlich D. (1997). RanBP1 is crucial for the release of RanGTP from importin beta-related nuclear transport factors. FEBS Lett 419 (2-3), 249–254. 10.1016/s0014-5793(97)01467-1 9428644

[B3] Boudhraa Z. Carmona E. Provencher D. Mes-Masson A. M. (2020). Ran GTPase: A key player in tumor progression and metastasis. Front. Cell Dev. Biol 8, 345. 10.3389/fcell.2020.00345 32528950 PMC7264121

[B4] Brooks B. R. Brooks C. L. 3rd Mackerell A. D. Jr. Nilsson L. Petrella R. J. Roux B., (2009). Charmm: The biomolecular simulation program. J. Comput. Chem 30 (10), 1545–1614. 10.1002/jcc.21287 19444816 PMC2810661

[B5] Costa M. G. S. Batista P. R. Bisch P. M. Perahia D. (2015). Exploring free energy landscapes of large conformational changes: Molecular dynamics with excited normal modes. J. Chem. Theory Comput 11 (6), 2755–2767. 10.1021/acs.jctc.5b00003 26575568

[B6] Dudas B. Merzel F. Jang H. Nussinov R. Perahia D. Balog E. (2020). Nucleotide-specific autoinhibition of full-length K-Ras4B identified by extensive conformational sampling. Front. Mol. Biosci 7, 145. 10.3389/fmolb.2020.00145 32754617 PMC7366858

[B7] Dudas B. Perahia D. Balog E. (2021). Revealing the activation mechanism of autoinhibited RalF by integrated simulation and experimental approaches. Sci. Rep 11 (1), 10059. 10.1038/s41598-021-89169-5 33980916 PMC8115643

[B8] Forwood J. K. Lonhienne T. G. Marfori M. Robin G. Meng W. Guncar G., (2008). Kap95p binding induces the switch loops of RanGDP to adopt the GTP-bound conformation: Implications for nuclear import complex assembly dynamics. J. Mol. Biol 383 (4), 772–782. 10.1016/j.jmb.2008.07.090 18708071

[B9] Gorlich D. Kutay U. (1999). Transport between the cell nucleus and the cytoplasm. Annu. Rev. Cell Dev. Biol 15, 607–660. 10.1146/annurev.cellbio.15.1.607 10611974

[B10] Gorlich D. Pante N. Kutay U. Aebi U. Bischoff F. R. (1996). Identification of different roles for RanGDP and RanGTP in nuclear protein import. EMBO J 15 (20), 5584–5594. 10.1002/j.1460-2075.1996.tb00943.x 8896452 PMC452303

[B11] Gorlich D. (1998). Transport into and out of the cell nucleus. EMBO J 17 (10), 2721–2727. 10.1093/emboj/17.10.2721 9582265 PMC1170612

[B12] Gur M. Madura J. D. Bahar I. (2013). Global transitions of proteins explored by a multiscale hybrid methodology: Application to adenylate kinase. Biophys. J 105 (7), 1643–1652. 10.1016/j.bpj.2013.07.058 24094405 PMC3791301

[B13] Guttler T. Madl T. Neumann P. Deichsel D. Corsini L. Monecke T., (2010). NES consensus redefined by structures of PKI-type and Rev-type nuclear export signals bound to CRM1. Nat. Struct. Mol. Biol 17 (11), 1367–1376. 10.1038/nsmb.1931 20972448

[B14] Jo S. Cheng X. Islam S. M. Huang L. Rui H. Zhu A., (2014). CHARMM-GUI PDB manipulator for advanced modeling and simulations of proteins containing nonstandard residues. Adv. Protein Chem. Struct. Biol 96, 235–265. 10.1016/bs.apcsb.2014.06.002 25443960 PMC4739825

[B15] Jo S. Kim T. Iyer V. G. Im W. (2008). CHARMM-GUI: A web-based graphical user interface for CHARMM. J. Comput. Chem 29 (11), 1859–1865. 10.1002/jcc.20945 18351591

[B16] Joseph J. (2006). Ran at a glance. J. Cell Sci 119 (17), 3481–3484. 10.1242/jcs.03071 16931595

[B17] Kurkcuoglu Z. Bahar I. Doruker P. (2016). ClustENM: ENM-based sampling of essential conformational space at full atomic resolution. J. Chem. Theory Comput 12 (9), 4549–4562. 10.1021/acs.jctc.6b00319 27494296 PMC5088496

[B18] Monecke T. Guttler T. Neumann P. Dickmanns A. Gorlich D. Ficner R. (2009). Crystal structure of the nuclear export receptor CRM1 in complex with Snurportin1 and RanGTP. Science 324 (5930), 1087–1091. 10.1126/science.1173388 19389996

[B19] Ohtsubo M. Okazaki H. Nishimoto T. (1989). The RCC1 protein, a regulator for the onset of chromosome condensation locates in the nucleus and binds to DNA. J. Cell Biol 109 (4 1), 1389–1397. 10.1083/jcb.109.4.1389 2677018 PMC2115805

[B20] Paci G. Caria J. Lemke E. A. (2021). Cargo transport through the nuclear pore complex at a glance. J. Cell Sci 134 (2), jcs247874. 10.1242/jcs.247874 33495357

[B21] Phillips J. C. Hardy D. J. Maia J. D. C. Stone J. E. Ribeiro J. V. Bernardi R. C., (2020). Scalable molecular dynamics on CPU and GPU architectures with NAMD. J. Chem. Phys 153 (4), 044130. 10.1063/5.0014475 32752662 PMC7395834

[B22] Renault L. Kuhlmann J. Henkel A. Wittinghofer A. (2001). Structural basis for guanine nucleotide exchange on Ran by the regulator of chromosome condensation (RCC1). Cell 105 (2), 245–255. 10.1016/s0092-8674(01)00315-4 11336674

[B23] Resende-Lara P. T. Costa M. G. S. Dudas B. Perahia D. (2022). Adaptive collective motions: A hybrid method to improve conformational sampling with molecular dynamics and normal modes. biorxiv [Preprint]. Available at: https://www.biorxiv.org/ .

[B24] Rudack T. Jenrich S. Brucker S. Vetter I. R. Gerwert K. Kotting C. (2015). Catalysis of GTP hydrolysis by small GTPases at atomic detail by integration of X-ray crystallography, experimental, and theoretical IR spectroscopy. J. Biol. Chem 290 (40), 24079–24090. 10.1074/jbc.M115.648071 26272610 PMC4591799

[B25] Scheffzek K. Klebe C. Fritz-Wolf K. Kabsch W. Wittinghofer A. (1995). Crystal structure of the nuclear Ras-related protein Ran in its GDP-bound form. Nature 374 (6520), 378–381. 10.1038/374378a0 7885480

[B26] Seewald M. J. Korner C. Wittinghofer A. Vetter I. R. (2002). RanGAP mediates GTP hydrolysis without an arginine finger. Nature 415 (6872), 662–666. 10.1038/415662a 11832950

[B27] Seki T. Hayashi N. Nishimoto T. (1996). RCC1 in the Ran pathway. J. Biochem 120 (2), 207–214. 10.1093/oxfordjournals.jbchem.a021400 8889801

[B28] Sun Q. Carrasco Y. P. Hu Y. Guo X. Mirzaei H. Macmillan J., (2013). Nuclear export inhibition through covalent conjugation and hydrolysis of Leptomycin B by CRM1. Proc. Natl. Acad. Sci. U. S. A 110 (4), 1303–1308. 10.1073/pnas.1217203110 23297231 PMC3557022

[B29] Vetter I. R. Arndt A. Kutay U. Gorlich D. Wittinghofer A. Kutay U. (1999a). Structural view of the Ran-Importin beta interaction at 2.3 A resolution. Cell 97 (5), 635–646. 10.1016/s0092-8674(00)80774-6 10367892

[B30] Vetter I. R. Nowak C. Nishimoto T. Kuhlmann J. Wittinghofer A. (1999b). Structure of a ran-binding domain complexed with ran bound to a GTP analogue: Implications for nuclear transport. Nature 398 (6722), 39–46. 10.1038/17969 10078529

