# Supplementary material for: Small GTPase Ran: Depicting the nucleotide-specific conformational landscape of the functionally important C-terminus
Source: Front Mol Biosci. 2023 Jan 16;10:1111574. doi: 10.3389/fmolb.2023.1111574 (PMC9885160; doi:10.3389/fmolb.2023.1111574)
Supplement: Supplementary file 4 [file Image1.pdf]

Supplementary Material

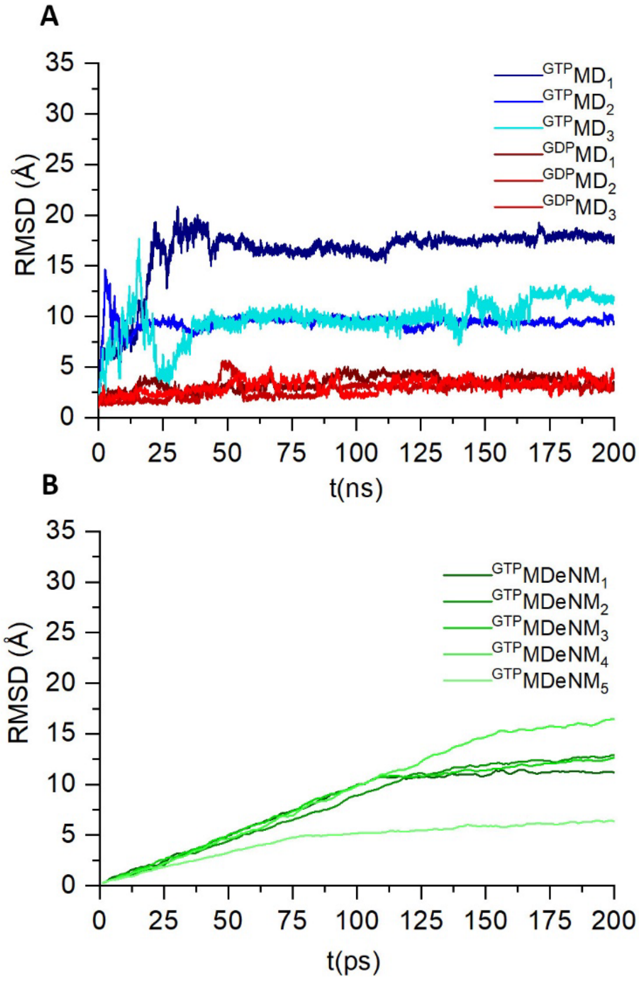

Supplementary Figure 1. Backbone RMSD in respect to the energy minimized solvated structures. (A) Ran-GDP (shades of red) and Ran-GTP (shades of blue); and (B) of Ran-GTP aMDeNM (shades of green) simulation.

Supplementary Movie 1. Ran-GDP MD simulation. Switch I is colored blue, Switch II red and the C-terminal purple.

Supplementary Movie 2. Ran-GTP MD3 simulation. Switch I is colored blue, Switch II red and the C-terminal purple.

Supplementary Movie 3. Ran-GTP aMDeNM simulation. Switch I is colored blue, Switch II red and the C-terminal purple.
